# Occurrence and prevalence of *Legionella* species in dental chair units in Germany with a focus on risk factors

**DOI:** 10.1007/s10096-023-04659-w

**Published:** 2023-09-12

**Authors:** Marleen Optenhövel, Alexander Mellmann, Thorsten Kuczius

**Affiliations:** Institute of Hygiene, University Hospital Münster, Robert Koch-Straße 41, 48149 Münster, Germany

**Keywords:** *Legionella*, *Legionella anisa*, Dental unit waterlines, Risk factors, Dental chairs

## Abstract

**Purpose:**

Water-bearing instruments and treatments in dental units produce aerosols originating from the dental unit waterlines (DUWLs), which are often microbially contaminated. Particularly, the presence of *Legionella* mainly realized as aerosols leads to a risk of infection in patients and dental staff.

**Methods:**

Here, we record the general bacteriological status of DUWLs in Germany and investigated the prevalence of *Legionella* spp., with a focus on identification and occurrence of distinct species considering the various aspects of dental practice such as dental chair equipment, disinfection methods, and temperatures.

**Results:**

Out of 3789 water samples of 459 dental practices, collected in the years 2019 and 2020, 36.4% were *Legionella* positive with predominance of *L. anisa* (97.89%) identified by MALDI-TOF biotyping. *L. pneumophila* was detected very rarely. Risk factor analysis revealed that temperatures >20°C are a significant factor for increased *Legionella* colonization.

**Conclusion:**

In order to minimize the risk of infection, routine monitoring of the water quality in dental chair units is recommended with regard to general microbiological loads and to the presence of *Legionella* as opportunistic pathogen as well as the regular application of routine disinfection procedures.

## Introduction

A good microbiological water quality is essential in dental unit waterlines (DUWLs) for human health and safety. Water pipes are flushed with potable water of the local water supplier that rinses the DUWLs, particularly chair units and instruments. Dentists and dental staff as well as patients are exposed to aerosols on a daily basis produced by medical water-bearing instruments as high-speed turbines, air polishing systems, hand pieces, and mechanical scalers [1–4]. Several studies reported about microbiological contaminations of DUWLs [5–10]. Sources of bacterial inputs include the water piped into the units and the use of bottled water systems as well as the suction of patients’ saliva into the line. Microorganisms adhere on the surfaces and consequently form biofilms, which may act as reservoir for waterborne, environmental, and man-made infections. Stagnation of the water for longer periods favors additional biofilm formation and cell number increase [11].

Microbiological loads can be minimized by the application of biocides [12] and chemical and physical disinfections [13, 14]. However, bacterial accumulations occur despite repeated decontamination processes [15–17]. Although the intensity of microbiological loads varies in different DUWLs, other environmental factors such as the chemical and microbiological water quality in the system, the temperature, the application of disinfections procedures, and the dentists’ units and chair models [16] may have an impact on the contamination level.

Therefore, regular monitoring is recommended with regard to general microbiological loads and in particular to the presence of *Legionella* as opportunistic pathogens [18]. *Legionella* exist ubiquitously in watery environments, particularly in urban water distribution systems, air conditioning devices and cooling towers [19–22] but in DUWLs as well [23, 24].

The genus of the rod-shaped Gram-negative bacteria comprises about 66 species with more than 70 serotypes [25, 26]. More than half of all species are able to cause infections to humans, mainly when taken up as aerosols into the lower respiratory tract [27]*. L. pneumophila* was found to be responsible for most of the reported cases of legionellosis but may also cause other clinical manifestations including Pontiac fever [27]. Although *Legionella* species differ in pathogenicity, the non-*pneumophila* species as *L. anisa*, *L. bozemanii*, and *L. longbeachae* also have high infectious potential to humans [28–30].

Even though few individual cases of *Legionella pneumophila* infections in dentists and patients after visits to dental practices have been reported so far [31, 32], the antibody prevalence against *Legionella* in dentists and dental staff is significantly higher compared with the general population [33, 34]. This observation indicated that aerosols generated in the practice are a source of loads with *Legionella* spp. A study on the microbiological contamination of DUWLs in various European countries showed a generally high microbiological contamination of every second unit, but only occasional findings of *L. pneumophila* [35].

Analyzing associations among microbiological contamination, the presence of *Legionella* in DUWLs, and the seropositivity of dental staff combined with an infection risk assessment, we recorded the *Legionella* and general bacteriological status of DUWLs in Germany in this study. We investigated the prevalence of *Legionella* spp. with a special focus on identification and occurrence of distinct species considering the various aspects of the dental practice such as dental chair equipment and disinfection methods.

## Materials and methods

### Sample collection and microbiological evidence

During a 2-year period from January 2019 to December 2020, water samples from dental chairs were collected from 459 dental practices in Germany. All practices were supplied with local municipal water that complies with the standards of the German Drinking Water Ordinance [36]. As known and reported by the dental practice, information was documented about the chair units (manufacturer and model) and the existence of a disinfection system based on oxidizing action such as chlorine and/or hydrogen peroxide to use the water as service water. In total, 3789 water samples taken from the air/water syringes or the spittoons were collected according to the Robert Koch Institute’s guideline for infection prevention in dentistry [18]. The water temperature was determined according to DIN 38404. The total microbiological numbers were counted on DEV agar (Xebios, Düsseldorf, Germany). Briefly, 1 ml of the water sample was mixed with agar prior to incubation at 36 °C for 48 h. The colony-forming units (cfu) indicating viable bacteria were counted and calculated as cfu per milliliter. For *Legionella* detection, a total volume of 1 ml of the water sample was applied to GVPC agar plates comprising buffered charcoal yeast extract with glycine, vancomycin, polymyxin B, and cycloheximide (Xebios, Düsseldorf, Germany). Plates were incubated in a box under moist atomsphere at 36 °C for 7 to 10 days followed by *Legionella* cfu counting.

### Confirmatory detection of Legionella species and identification

Single colonies suspected to *Legionella* species were cultured on buffered charcoal yeast extract agar (BCYE; Xebios) for confirmation while growth on Columbia blood agar plates (Oxoid, Wesel, Germany) was absent*. Legionella* spp. were differentiated by serotyping using commercial antisera (Legionella Latex Test; Oxoid) carried out on the basis of the manufacturer’s instructions. The test sera indicated the presence of *L. pneumophila* serogroup (SG) 1, *L. pneumophila* SG 2–14, and *Legionella* non-*pneumophila* recognizing *L. bozemanii*, *L. longbeachae*, *L. dumoffii*, *L. gormanii*, *L. jordanis*, *L. micdadei*, and *L. anisa*. In addition, colonies were differentiated with regard to morphology and fluorescence properties under UV light at 320 nm.

The *Legionella* spp. was assigned by MALDI-TOF biotyping (Bruker Daltonik, Bremen, Germany). Single colonies were extracted by ethanol-formic acid, directly inoculated on the target, and overlayed with a cyano-4-hydroxycinnamic acid matrix (Sigma-Aldrich, Germany) according to the manufacturer’s protocols. The spectrum of each isolate was matched along the spectra library (database). The best matches were generated with confidence scores at which a score of > 1.7 and < 2.0 was considered genus and > 2.0 species level. The reference strains *L. pneumophila* SG1 (ATCC33152), *L. pneumophila* SG5 (ATCC33737), *L. longbeachae* (ATCC33462), and *L. anisa* (DSM17627) were used as controls based on the American Type Culture Collection (ATCC) and originated from the German Collection of Microorganisms and Cell Cultures (Leibniz Institute DSMZ, Braunschweig, Germany).

### Statistical analysis

**Fig. 1:**
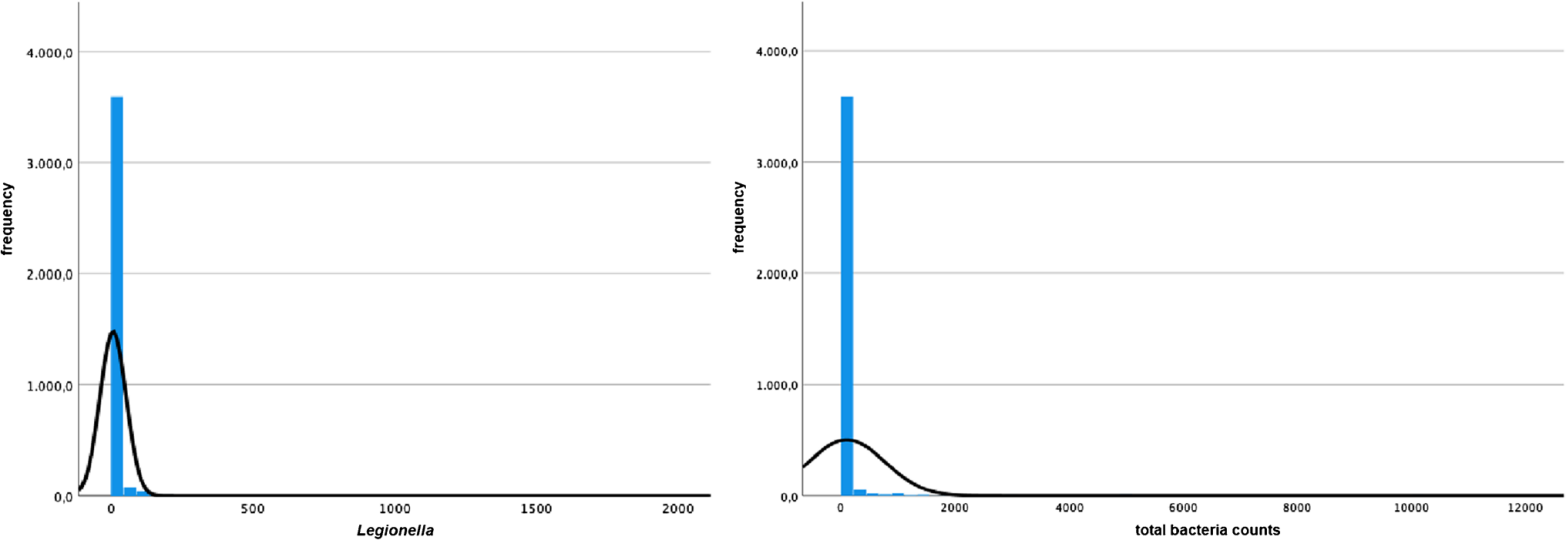
Histograms of the distributions (black lines) of the variable *Legionella* and of the variable total bacteria counts, each given in cfus/ml, plotted versus frequencies (colums)

Statistical analysis was carried out using IBM SPSS statistics 27 (IBM, Armonk, New York). The data were analyzed using descriptive statistics and nonparametric tests because both the Kolmogorov–Smirnov test and the graphical analysis revealed that the dependent variables (*Legionella* cfu/ml and total bacteria count as cfu/ml) were not distributed normally (D(3760 = 0.425, *p* = 0.000; D(3782) = 0.439, *p* = 0.000; Fig. 1). Descriptive statistics were calculated for the total bacteria count, *Legionella* counts/ml, and the different *Legionella* species. Samples were grouped by temperature with a separation value of 20 °C because at lower temperatures, *Legionella* were only able to multiply very slowly, if at all. Afterwards, a Mann–Whitney *U* test was conducted to compare the *Legionella* contamination between these groups. In order to detect a possible influence of the disinfecting agent used on the *Legionella* cfu/ml, a Kruskal–Wallis test was conducted, testing for between-group differences between hydrogen peroxide (H_2_O_2_) and a combination of H_2_O_2_ and chlorine (Cl_2_). The Kruskal–Wallis test is the nonparametric alternative of a one-way ANOVA and is rank based, meaning that it sorts all values according to size. The lowest value is assigned to rank 1 and the highest value is assigned to the highest rank. For each group, the mean rank is calculated and compared, with a higher mean rank indicating higher values in a group. In the next step of the analysis, four additional Kruskal–Wallis tests were performed to test for a possible influence of the manufacturer of the dental chair unit and the dental chair unit models on the *Legionella* count/ml and the total bacteria count. Manufacturers and dental chair unit models were excluded from the analysis if *N* was ≤ 20. If one of the Kruskal–Wallis tests was significant, a Dunn post hoc test was done to conduct a pairwise comparison of all groups in order to uncover the significant between-group differences. To prevent alpha error cumulation, Bonferroni correction was conducted. A binary logistic regression analysis was performed to evaluate the influence of the independent variables total bacteria count, disinfecting agent, temperature, and the dental chair unit manufacturers on the risk of a *Legionella* contamination. A likelihood ratio test was conducted in order to test the overall fit of the model, while the goodness of fit was examined using the Hosmer–Lemeshow test.

## Results

In this study, water samples of 459 dental practices were analyzed routinely for presence of *Legionella* spp. In total, out of 3789 water samples, 2412 samples were negative (63.6%) while 1377 (36.4%) were tested positive for *Legionella* presence. The *Legionella* counts as colony-forming units (cfus) on plates varied immensely in the individual water samples with 1 to 1850 cfu/ml (median 6.00 (IQR = 2–21)). Isolates with assignment to *Legionella* species grew on selective plates but not on Columbia blood agar plates. Interestingly, a very predominant proportion of the isolates showed a uniform morphology of identical appearance. Colonies showed round appearance with smooth edge coloring in white to light gray. This type of colony fluoresced under UV light in radiant form. Isolates, identified on species level by MALDI biotyping, were determined as *L. anisa*. Serotyping resulted in reactions only with the polyvalent serum. In contrast, *L. pneumophila* isolates agglutinated with the sera of groups 1 or 2–14. These microorganisms showed a large and shapeless colony form with speckled gray-green iridescence, and frosted glass-like appearance and mirror shape look without fluorescence properties.

*L. anisa* was the predominant species in almost all cultivations in dental chair units of the analyzed water samples. *L. pneumophila* were identified exclusively in 29 samples (2.11%) or additionally as a second species in 36 samples (2.61%). Other *Legionella* species were not detected (Table 1).

**Table 1:** Occurrence of *Legionella* spp. in dental unit waterlines

*Legionella*	*N*	%
*L. anisa*	1312	95.28
*L. anisa* and *L. pneumophila*	36	2.61
*L. pneumophila*	29	2.11
Total	1377	100

While the preceding analyses were focused on the detection and classification of *Legionella*, we investigated on factors influencing the occurrence of this microorganism such as the number of the accompanying flora, the water temperature in the practice, the application of disinfecting agents and the disinfection processes as well as the dental chair manufacturer and the respective model.

Regarding the total bacterial count, 37.8% of all samples featured a total bacteria count > 1 cfu/ml (median 7.00 (IQR = 2–56)). The numbers of cfus differed highly in the individual water samples and varied from 1 to 10,000 (Table 2). However, due to the result of the binary logistic regression, the *Legionella* presence and the total bacteria count did not correlate.

**Table 2:** Total bacteria counts and *Legionella* presence in DUWLs

Variable	*N*	Median	Min	Max
*Legionella* (cfu/ml)	3760	6.00	1	1850
< 20 °C	1927	0	0	820
> 20 °C	1674	0	0	1850
Total bacteria count (cfu/ml)	3782	7.00	1	10,000

The temperature may have a predominant impact on the microbial survival and multiplication rate. We differentiated the detected number of *Legionella* cfu/ml into samples with water temperatures below and above 20 °C, when known. As expected, higher numbers of *Legionella* cfu/ml were found in samples with temperatures ≥ 20 °C (mean rank: 1853.40) compared with samples < 20 °C (mean rank: 1755.48) at a high significance level (*U* = 1,525,188.00, *Z* =  − 3.365, *p* = 0.001), calculated using the Mann–Whitney *U* test (Table 2).

Using regular disinfections, operating water in dentists’ chairs is mixed with hydrogen peroxide, and on chlorine basis is often additionally added to the water supply to the dental chair, e.g., by using bottle systems. The use of the disinfections processes was analyzed with regard to microbiological survival. As known, 3085 water samples were treated additionally with chlorine, while 50 samples contained only hydrogen peroxide. For the remaining samples (*N* = 681), no information about use of disinfecting agents were provided; thus, these samples were not considered in the analysis. In total, no significant differences were found between the two groups of disinfections regarding *Legionella* contamination (Kruskal–Wallis test; H(2) = 0.573, *p* = 0.751).

To analyze an effect of dental chair units from the various manufacturers on *Legionella* contamination, 16 manufacturers met the inclusion criteria of *N* > 20. Significant differences were found between the manufacturers XO and Sirona, Ultradent and SternWeber as well as KAVO and Planmeca (Table 3). The *Legionella* contamination as well as the total microbiological counts of XO (Table 4) was the lowest compared with the other manufacturers.

**Table 3:** Results of *Legionella* and total bacteria count contamination regarding dental chair units based on Kruskal–Wallis and Dunn post hoc tests

Test	*Legionella* (cfu/ml) with regard to					Total bacteria count with regard to				
	Dental chair unit manufacturer		Dental chair unit model			Dental chair unit manufacturer			Dental chair unit model	
KWT	H	*p*		H	*p*		H	*p*	H	*p*
	46.58	.000		47.89	.000		38.73	.001	38.73	.001
DPH	Z	*p*		Z	*p*		Z	*p*		
XO* Sirona	314.89	.014	KAVO 1058* C5 +	177.65	.001	xo* Sirona	304.36	.023		
XO* Ultradent	403.11	.001	KAVO 1058* C8 +	− 196.89	.000					
XO* SternWeber	403.11	.005	Sinius* C5 +	150.29	.026					
KAVO* Planmeca	− 116.47	.022								

KWT Kruskal–Wallis test, DPH Dunn post hoc test

**Table 4:** Presence of *Legionella* and total bacteria count in regard with dental chair unit manufacturers

Manufacturer	*Legionella*				Total bacteria count			
	*N*	Max	Mean rank	Percentile 75th	*N*	Max	Mean rank	Percentile 75th
Anthos	68	70	1221.35	1	69	1000	1256.86	1
Belmont	42	90	1242.30	1.25	42	400	1373.99	4.25
Castellini	45	256	1307.51	2	45	173	1365.83	3.50
DKL	63	82	1245.02	1	63	106	1114.17	0
F1	23	149	1328.65	7	23	452	1094.28	0
Finndent	73	100	1150.16	0	73	864	1294.33	2.75
Hekadental	37	45	1055.43	0	37	278	1250.26	1.50
KAVO	624	211	1234.36	1	624	1000	1221.82	1
Planmeca	97	167	1350.96	3.50	97	1500	1381.04	8
Ritter	27	113	1178.06	0	27	191	1094.69	8
Sirona	925	1850	1315.18	2	925	7100	1332.15	2
SternWeber	73	97	1443.94	5	73	168	1395.43	5
TGA	43	55	1304.78	3	43	412	1333.47	3
Thomas	96	19	1251.38	2	96	196	1297.43	2.75
Ultradent	270	400	1404.42	5	270	4800	1295.43	2
XO	62	12	1000.82	0	62	112	1027.79	0
Total	2568	1850			2569	7100		

The minimum, 25th, and the 50th percentiles were always 0 and not included in the table

*specified manufacturers or models in direct comparison with each other

Furthermore, we analyzed the correlation of *Legionella* presence and dental chair models; only 13 of the dental chair unit models met the *N* > 20 criteria. Significant differences were found between KAVO 1058 and Sirona C5 + , KAVO 1058 and Sirona C8 + , and Sirona Sinius and Sirona C5 + (Tables 3 and 5). Based on our data, the KAVO 1058 model had a significantly lower *Legionella* colonization compared with the Sirona models C5 + and C8 + . Taken together, statistical analyses resulted in significant differences between both the dental chair unit manufacturers and the dental chair unit models. A relationship between the dental chair unit manufacturers and the total bacteria count indicated significant results while the post hoc test showed only one significant between-group difference between XO and Sirona in favor for XO (Tables 3 and 4). In contrast, only the Kruskal–Wallis test indicated significant differences between the different dental chair unit models, while the post hoc analysis failed in significant between-group differences (Tables 3 and 5).

**Table 5:** Descriptive statistics of the *Legionella* cfu/ml and total bacteria count cfu/ml for dental chair unit models

Dental chair unit	Numbers	*Legionella* cfu/ml			Total bacteria count cfu/ml	
		Mean rank	Percentile		Mean rank	Percentile 75th
			50th (Median)	75th		
KAVO 1058	133	420.85	0	0	438.63	1
KAVO E80	31	456.85	0	1	411.15	0
Sirona C1 +	23	485.07	0	2	457.20	2
Sirona C2	41	551.91	0	9	548.87	14.50
Sirona C2 +	69	528.01	0	3	523.94	3
Sirona C4 +	31	456.39	0	1	412.31	1
Sirona C5 +	47	597.15	1	8	535.80	10
Sirona C8 +	28	616.16	1	7	448.52	1
Sirona Integro	55	477.43	0	1	480.11	2
Sirona M1	205	509.38	0	2.25	500.38	2
Sirona M1 +	88	517.44	0	3	525.81	7
Sirona Sinius	123	448.00	0	0	525.44	5
Sirona Teneo	103	452.55	0	1	456.98	2
Total	977					

The 25th percentile was always 0, same as the median for total bacteria count, and are therefore not displayed in the table

While the preceding analyses focused on different parameters affecting the level of *Legionella* contamination, the previous tested factors were combined now to a binary logistic regression model (Table 6). The aim of this analysis was to assess the extent to which each factor may contribute to an increased or decreased risk of *Legionella* presence. The results of the likelihood ratio test proved that the inclusion of the independent variables improved the overall fit of the model significantly (*χ*^2^(18) = 63.677, *p* = 0.000), and the outcome of the Hosmer–Lemeshow test indicated a good fit of the data to the model (*χ*^2^(8) = 13.900, *p* = 0.084). Based on four independent variables (temperature, total bacteria count, presence of disinfecting agents, and dental chair unit manufacturer), the binary logistic regression analysis proved only significant results for the temperature and the dental chair unit manufacturer. Regarding temperature, the risk of *Legionella* presence was up to 28.8% lower when the temperature was < 20 °C. In the preceding analyses, XO was the manufacturer with the lowest *Legionella* contamination in our hands; therefore, XO was used as reference category (Table 6). Nine out of fifteen different dental chair unit manufacturers showed an enhanced risk of *Legionella* colonization with an odds ratio > 1.000 at a significance level > 0.05. SternWeber showed the highest increase (400.4%) and KAVO was the one with the lowest (154.2%) (Table 6). Overall, factors found to increase the risk of the *Legionella* spp. presence included temperature > 20 °C as well as dental chair units from nine different manufacturers (marked with * in Table 6).

**Table 6:** Results of the binary logistic regression analysis

Predictor	β	*SE* β	Wald’s *χ*^2^	*df*	*p*	*e*^β^ (odds ratio)	Increased risk (%)	95% Confidence interval
Total bacteria count	.000	.000	.992	1	.319	1.000	0	[1.000; 1.001]
Disinfecting agent	.022	.329	.005	1	.945	1.023	2.3	[.537; 1.948]
Temperature < 20 °C	− .340	.091	14.080	1	.000*	.712	28.8	[.596; .850]
DCU manufacturer			41.071	15	.000*			
Anthos	.899	.467	3.706	1	.054	2.456	145.6	[.984; 6.132]
Belmont	1.028	.506	4.077	1	.043*	2.796	179.6	[1.031; 7587]
Castellini	1.194	.500	5.702	1	.017*	3.302	230.2	[1.239; 8.801]
DKL	.977	.472	4.281	1	.039*	2.656	165.6	[1.053; 6.699]
F1	1.106	.583	3.595	1	.058	3.021	202.1	[.963; 9.473]
Finndent	.586	.474	1.530	1	.216	1.797	79.7	[.710; 4.548]
Hekadental	.017	.586	.001	1	.977	1.017	1.7	[.322; 3.209]
KAVO	.933	.394	5.613	1	.018*	2.542	154.2	[1.175; 5.499]
Planmeca	1.124	.440	6.533	1	.011*	3.077	207.7	[1.300; 7.285]
Ritter	.948	.618	2.353	1	.125	2.580	158.0	[.768; 8.665]
Sirona	1.140	.390	8.561	1	.003*	3.127	212.7	[1.457; 6.712]
SternWeber	1.610	.453	12.612	1	.000*	5.004	400.4	[2.058; 12.170]
TGA	1.226	.502	5.978	1	.014*	3.408	240.8	[1.275; 9.109]
Thomas	− .096	.516	.035	1	.852	.908	90.8	[.331; 2.495]
Ultradent Xo (reference category)	1.326 NA	.405 NA	10.694 NA	1 NA	.001* NA	3.765 NA	276.5 NA	[1.701; 8.333] NA

Significant results are marked with *

NA not applicable

## Discussion

Microorganisms often contaminate DUWLs with the consequence of biofilm formation [24, 37]. Monitoring of the total bacteria counts and the presence of *Legionella* is recommended by the Robert Koch Institute’s guideline for infection prevention in dentistry [18] in order to minimize the risk of infection of patients and dental staff. *Legionella* contaminations are often reported with a focus on the prevalence of *L. pneumophila* [37–39] and on differentiation between *L. pneumophila* and *L.* non-*pneumophila* [15, 40–42]. Our study aimed to the general detection of *Legionella* in DUWLs following differentiation into species level using the MALDI-TOF biotyping technique. Out of 3789 dental chair water samples taken from 459 dental practices in Germany, approximately one third (36.4%) were tested positive for *Legionella* presence. Another study demonstrated only slightly lower contamination rates with 27.8% *Legionella*-positive samples out of 22 dental practices in Germany [15]. Out of the *Legionella*-positive samples, 28% were assigned to *L. pneumophila* serogroup 1, despite no differentiated species distinction. At an institute with 50 dental chair units in Olomouc (Czech Republic), *L. pneumophila* serogroup 4 dominated in the DUWLs, which were colonized with *L. anisa* and *L. quateirensis* as well [37]. Our data provided rare evidence of *L. pneumophila* presence. Interestingly, on the level of species differentiation, *L. anisa* was predominant in almost all samples of DUWLs, especially detected as pure culture and rarely associated with other *Legionella* species. Only very few water samples carried *L. pneumophila* exclusively (2.11%) or additionally (2.61%). *L. anisa* was isolated from humans [43]. Several studies reported about human infections developing pulmonary and extrapulmonary diseases [28, 29, 43–48], yet questioning the relevance of *L. anisa* as a health-threatening pathogen as most studies were case reports. Although of moderate to low pathogenicity to humans, it is assumed that this species may be infectious mainly for immunocompromised patients [49]. In the environment, the species is mainly found in hospital water systems and cooling tower waters [50, 51].

To identify first clues and conditions that favor a contamination and long-term survival of *L. anisa* in the DUWLs, we analyzed the data with regard to total cell counts, the water temperature, the disinfection impacts, and the chairs and models of unit manufacturers.

Temperatures below 20 °C limited and inhibited *Legionella* growth while multiplication occurred at higher temperatures up to approximately 50 °C, and the reproduction is encouraged when water stagnates [52]. Our data show a significantly lower *Legionella* contamination level in samples with temperatures < 20 °C compared with those > 20 °C. The data are consistent with the results of the binary logistic regression analysis stating that the risk of a *Legionella* contamination is 28.8% lower in samples < 20 °C. This finding concurred with *L. pneumophila* results published earlier [53]. *Legionella* contamination and the total bacteria count showed no significant correlation.

Based on information of the practices, most of the DUWLs were treated with hydrogen peroxide and additionally with chlorine, wherein there was no correlation of *Legionella* contamination and the disinfecting agents. These results can only be evaluated to a limited extent as of less information regarding use, frequency, and concentration of the disinfecting agents and rinsing protocols as well as due to the fact that nearly all dental chairs are routinely treated with various disinfections. In general, considerably high concentrations of hydrogen peroxide (1000 µg/ml) are necessary to achieve the same reduction level of *L. pneumophila* as with chlorine at 1 µg/ml [12].

Dental chair units from different manufacturers were rarely compared with each other in terms of bacterial loads. Colonization of Sirona and KAVO chair units was compared directly by either with a limited number for statistical evaluation [54] or significant colonization count differences that were proven among manufacturers [55]. One study reported about higher *Legionella* counts in KAVO models compared with Sirona, but with the note that all KAVO units were located at the same department of the university hospital and the potable water fed into the units had increased *Legionella* counts [56]. In direct comparison of the dental chair units from various manufacturers in our study, XO statistically had the lowest *Legionella* and total bacteria counts. Hence, the water quality of both the supplier as well as the distribution in the building and the dental unit are crucial factors for increased or minimized bacteriological colonization. This aspect limits the generalizability of our results because no data were available about the general water quality in the dental practices localized all over Germany.

Other impacts on bacterial loads are the equipment and the materials used in the dental chair unit. *L. pneumophila* adhered more efficiently on hydrophobic materials like polyvinylchloride (PVC) and galvanized steel than on stainless steel and glass [57]. Roughness of surfaces, ages of the chairs, and frequencies of use affect the efficiency of bacterial and *Legionella* colonization [58].

In total, our findings indicate a high incidence of *L*. non-*pneumophila* species with a colonization rate of approximately one third of all DUWLs despite application of disinfections. This occurrence may be underestimated as many existing test systems are primarily directed toward the detection of *L. pneumophila* neglecting and omitting species differentiation [31]. Yet, dentists and dental staff have increased antibody levels to *Legionella* compared to the general population [33, 34]. This seropositivity indicated the presence of *Legionella*-positive aerosols generated in a dental practice.

The reasons for *L. anisa* dominance in DUWLs will and may be very diverse, especially despite disinfection procedures. The analyzed parameters such as temperature and chair models did not allow a clear conclusion so that physiology and behavior of *L. anisa* should be taken into account with regard to growth conditions, increased tolerance formation to disinfections, formation of and embedding in a biofilm, and the microbiological ecology of the accompanying flora in DUWLs.

From the practical perspective and according to the recommendations of the Robert Koch Institute in Germany [18], the units should be rinsed for at least 2 min in the morning before practical treatments begin and for 20 s before each patient treatment. To minimize the risk of retrograde contamination, the units can be rinsed again at the end of the day. The water fed into the treatment unit complies with the drinking water ordinance. It is low in microorganisms, but not bacteria free. Therefore, new waterborne microorganisms will be added to the system as well, which represents a health risk for vulnerable patients such as immunocompromised persons and an infection risk in high-risk areas, such as in dental surgery, for contamination of open wounds and consequently for infection of the patient with bacteria-contaminated water. For invasive procedures and for high-risk patients, the use of sterile water delivery systems is recommended [59] because the use of upstream and terminal filters provides bacteria-free water. For practical use, sterile media can be used for rinsing and cooling [60].

To ensure the required drinking water quality in treatment units, the units have integrated disinfection systems, whereby disinfectants are continuously added to the service water. With regular use, this process prevents the increase of bacterial counts and subsequently an increased formation of biofilms. However, the chemical water additives approved by the manufacturer are not alone able to ensure safe sterility. It can be assumed that the chemical disinfectants only attack biofilms, especially old biofilms in old chair units, but do not remove them. In general, biofilms can lead to impairments of the dental unit and potential health risks for patients. Moreover, the shelf life of the disinfectants and their compatibility with other chemical additives that may be applied to the water system in addition and at the same time should be checked regularly for their effect and action. With intermittent applications, microorganisms can develop resistances, so regular use should be seen as a prerequisite for low-loaded water [61]. In addition, changing a disinfectant with effects on different molecular levels can significantly minimize the development of resistances.

A relatively high ambient and room temperature can lead to growth of microorganisms. To minimize the risk of infection for patients and for the dental staff, the stagnation time of the water in the dental chair should be kept as short as possible and DUWLs may be rinsed frequently. Water that is used to treat or rinse patients’ teeth and mouths need not be pre-heated prior to use so that bacterial growth can be avoided or reduced due to the higher temperatures. Water temperatures lower than room temperature would even be desirable.

Thermal processes with water rinsing at > 60 °C (inactivation of *Legionella*) are also conceivable, but most hoses and connections in dental units are not heat stable. The material properties of the water-carrying components of a dental unit are of great importance. Biofilm formation mainly takes place in silicone hoses that connect the units to the working instruments. The manufacturer could consider the development of more interchangeable components here. In this way, the water-carrying hoses could be replaced at regular periods. In general, the tap water installation and the dental unit should be physically separated, and a free outlet of the unit is a weak point from a hygienic point of view [60]. Careful and strict applications of the dental staff according to the hygiene guideline support infection prevention [60].

In conclusion, more than one third of the examined dental chair units proved *Legionella* contamination exceeding the legal limit, so that dentists, staff, and patients are exposed to an increased risk of infection because of water aerosolization used in daily handling procedures. Out of numerous DUWL samples in Germany, interestingly *L. anisa* proved the predominant *Legionella* contaminant, while *L. pneumophila* was rare. The reason for this species-specific presence is still unknown, but the temperature above 20 °C evidenced as a significant factor for increased *Legionella* spp. colonization. Other impacts as materials and surfaces, disinfection changes and rinsing intervals, and the frequency of use of the manufacturer’s chairs as well as water quality and stagnation duration may also play a role for *Legionella* colonization.

## Data Availability

The authors confirm that the data supporting the findings of this study are available within the article.
